# A missed opportunity for public health: How impact assessment shaped EU rules on the marketing of unhealthy commodities to children

**DOI:** 10.1016/j.ssmqr.2023.100369

**Published:** 2024-06

**Authors:** Kathrin Lauber, Eleanor Brooks

**Affiliations:** Global Health Policy Unit, University of Edinburgh, UK

## Abstract

**Background:**

The 2015-2018 revision of the European Union's Audiovisual Media Services Directive, which governs the marketing of alcohol and unhealthy food to minors, failed to align with international best practice. Previous research has explained this ‘missed opportunity’ with reference to deficient political will, difficulties advocating for health, and industry pressure. We explore another explanation: the role of the impact assessment (IA) process in shaping decision-making.

**Methods:**

We first conducted an in-depth comparison of three versions of the IA report, employing qualitative content and framing analyses to establish *what changed* in the substantive content, framing, and evidence cited. Second, we used process-tracing, a qualitative method drawing on multiple data sources, to explore causal mechanisms, to assess *why these changes occurred*. Data sources include policy documents published proactively and obtained through access-to-document requests.

**Findings:**

Previously unpublished versions of the IA report show that stronger rules on advertising were preferred early in the policy process but later abandoned, and that concern for ‘balancing’ consumer protection and competitiveness shifted to focus on the latter. Following review by the Regulatory Scrutiny Board, a revised IA report narrowed the policy options, removing a requirement for member states to prevent childrens' exposure to alcohol advertising. Consequently, decision-makers were provided with an IA that did not offer adequate information on available measures to protect children.

**Interpretation:**

Changes made during the IA process, which determines the policy options presented to decision-makers, side-lined health concerns. We argue that engaging with the institutional structures which shape decision-making is crucial for those working to further public health.

## Highlights

• Provided with an opportunity to revise rules on marketing to kids, the EU maintained the status quo.
• We analyse the role of impact assessment using previously unpublished documentation.
• Our findings show a side-lining of health concerns following a strong initial position.
• We offer new insights into the ‘black box’ of EU policymaking and a more robust account of the AVMSD case.

## 1 Introduction

Reducing exposure to marketing – particularly for children – is widely acknowledged as an important step towards reducing the consumption of health-harming products (World Health Organization, 2010a, 2010b, 2012, 2022). While tobacco marketing is subject to comprehensive restrictions at European Union (EU) level, action on the marketing of other harmful products lags behind international best practice (Garde, 2020). The World Health Organization (WHO) has called for the regulation of marketing for high fat, sugar, and salt (HFSS) foods and alcohol since the early 2010s (WHO Regional Office for Europe, 2012; World Health Organization, 2010a, 2010b). Yet, when provided with an opportunity to update EU rules, EU institutions concluded a three-year process in 2018 with a decision against substantial changes. This came despite concerted efforts by the public health community to alert decision-makers to evidence of the adverse health impacts of exposure to unhealthy food and alcohol marketing for children, in particular, and the limits of existing provisions (European Public Health Alliance, 2016; European Public Health Alliance et al., 2017; University of Liverpool & European Heart Network, 2015). This ‘missed opportunity’ has been variously explained with reference to an absence of political will (Bartlett & Garde, 2017a), the European Commission's “dogmatic belief in the virtues of self-regulation” (Bartlett & Garde, 2017a, p. 390), difficulties in advocating for health within non-health committees in the European Parliament (Burrows, 2017), and industry efforts to prevent regulation (Bartlett & Garde, 2013). Yet, existing analyses largely omit consideration of how the draft text presented to legislators, which determines the policy options open to discussion and decision, was prepared. In this paper, we unpick the ‘black box’ of EU agenda-setting, using previously unpublished documentation and focusing on the impact assessment process, to assess how and why health concerns were marginalised.

At EU level, alcohol and HFSS food marketing are addressed in the Audiovisual Media Services Directive (AVMSD). These rules are supplemented by voluntary initiatives, including the EU Pledge, via which food and beverage companies commit to curb their advertising to children under the age of 12 (Bartlett & Garde, 2017b). Originally adopted in 2007 as a successor of the 1989 Television Without Frontiers Directive, the AVMSD regulates all broadcast and on-demand television services, with the overarching aims of enabling the provision of cross-border services whilst protecting cultural diversity, children and consumers, media pluralism, and the independence of national media regulators (Garde, 2010). Audiovisual commercial communications – covering television advertising as well as broader marketing techniques such as sponsorship and product placement – is one of many areas covered by the Directive.

Starting in 2015, the AVMSD was revised as part of the Commission's REFIT (Regulatory Fitness and Performance) programme. REFIT is designed to ensure that EU legislation remains ‘fit for purpose’ and, in the case of AVMSD, the revision sought to redress the lighter regulatory regime which had evolved for newer, on-demand media services. After the Commission proposed a legislative draft in May 2016, negotiations within and between the European Parliament and Council of the EU resulted in the adoption of a revised Directive in 2018. To the disappointment of the public health community, neither the legislative proposal nor the final law goes far beyond the original AVMSD regarding health (Bartlett & Garde, 2017a, 2017b; Garde, 2020). Small changes welcomed by public health groups include new attention to reducing children's *exposure* (as compared to the previous focus on not *targeting* children), and an explicit reference to the WHO Regional Office for Europe's nutrient profiling model in the Directive's preamble. However, rather than recommending statutory regulation or substantively strengthening rules, the revised Directive merely endorses a strengthening of self- and co-regulation – such as the EU Pledge – to reduce minors' exposure to alcohol and HFSS advertising.

REFIT is part of the *Better Regulation* agenda which guides EU policymaking, and particularly the actions of the executive, the European Commission. Though not legally binding, the agenda fundamentally determines what constitutes ‘high-quality’ regulation and regulatory processes. At its core, Better Regulation posits that policy should be minimally burdensome to citizens and businesses, evidence-based, and developed in consultation with stakeholders. These norms are implemented through a set of concrete tools: ex-ante impact assessment (IA), ex-post evaluation, and stakeholder consultation, supported by quality control and forward planning mechanisms. While drawing on a seemingly neutral notion of regulatory quality, programmes such as Better Regulation are neither objective nor value-free (Lauber & Brooks, 2023). Their potential to shape policy dynamics is exemplified by well-documented efforts of regulated industries, such as tobacco and chemicals, to influence the rules underpinning the EU's IA regime in ways that make it harder to pass health or environmental regulation (Smith et al., 2010, 2015).

The IA process is of particular interest, as it weighs and compares policy options based on their economic, social, and environmental impacts, and is meant to provide a neutral overview of the policy problem and available solutions. The department responsible for audiovisual media services within the Commission is the Directorate-General for Communications Networks, Content and Technology (DG Connect). DG Connect's legislative proposal for a revised AVMSD was informed by a parallel REFIT evaluation and IA, supported by extensive stakeholder consultation. A deeper look into this process, however, reveals a discrepancy: even though mandatory restrictions on HFSS and alcohol advertising were positioned as an option in early documentation, the final, published IA report – which presents the evidence underlying the Commission proposal – suggests that such measures were not assessed. This is particularly perplexing given that this process unfolded amidst sustained calls from public health advocates and researchers to strengthen protections, and substantial evidence for the health harms of alcohol and HFSS food marketing (Anderson et al., 2009; Cairns et al., 2009; Smith & Foxcroft, 2009). The IA report for the AVMSD revision underwent review by the Regulatory Scrutiny Board (RSB), a quality control body without whose positive opinion initiatives do not normally proceed. Documentation accompanying the proposal noted that the RSB first issued a *negative opinion* in March 2016, prompting a revision and resubmission of the IA. This revised version then received a *positive opinion with reservations* in April 2016 and underwent further changes before publication in May 2016. In this paper we use the previously unpublished draft IA reports as a starting point to investigate how and why health was side-lined in the revision of EU audiovisual marketing rules.

## 2 Material and methods

Adopting a critical lens, we approached policy documents such as impact assessment (IA) reports as narrative artefacts that *tell a story* about societal problems and appropriate solutions, doing discursive work to justify (non)decisions in the eyes of stakeholders, other political institutions, and citizens (Radaelli et al., 2013). After familiarising ourselves with the relevant, publicly available policy documents, we used requests under the EU access-to-documents regime to obtain (1) draft versions of the IA report for the revised AVMSD and (2) meeting minutes and correspondence related to the revision process (Appendix A). The core documents used in this study – 299 pages total – are listed in Appendix B. Though we sought to supplement and triangulate this data with accounts from those involved, repeated requests to interview relevant Commission officials were denied or went unanswered. Future research might seek to address this gap.

We analysed the available data – focusing on material relevant to the topics of HFSS and alcohol advertising – using three different approaches. First, we sought to establish *what* had changed during the revision of the IA reports, using a qualitative framing analysis to establish how (non)problems and (non)solutions were discussed and justified. The lead author coded all versions of the IA report using a framing matrix adapted from Jenkin et al. (2011), and the second author read all IA reports in-depth and coded one version, which was used as a basis for discussion of any discrepancies and corresponding refining of the analysis. Identifying changes across IA reports required a detailed comparison between versions, as edits were not signposted. Second, we conducted a content analysis of the evidence and stakeholder input cited in support of claims which relate to HFSS and alcohol marketing across the three versions of the IA report (details in Appendix C), to explore the role of evidence in shaping and justifying the assessment of policy options. Evidence, for this purpose, was approached in a broad sense to capture all sources cited in support of relevant statements. An initial analysis by the lead author was reviewed by the second author, and cases where classification was challenging were resolved jointly. Third, and drawing on broader documentation of the policy process, we used process tracing (Beach & Pedersen, 2019), a qualitative method focused on identifying and testing causal mechanisms, to explore what may explain the observed changes. To do so, we developed three plausible mechanisms based on relevant (grey and academic) literature and coverage of the AVMSD process, and assessed the available data to identify evidential ‘fingerprints’ which support or weaken confidence in each mechanism (see Appendix D for details).

In line with wider efforts to maintain high reporting standards for qualitative research (O'Brien et al., 2014), a fuller description of the stages of our analysis can be found in the appendices.

## 3 Results

Our analysis of the previously unpublished first draft IA report from 22 February 2016 shows that stronger rules on alcohol advertising were not only assessed but included in the ‘preferred option’, the Commission's policy recommendation and basis for the legislative proposal (no strengthened rules were included for HFSS advertising). Following a negative RSB opinion on the draft IA on 18 March 2016, a revised draft was resubmitted by DG Connect only seven days later. In this short timespan, the option to tighten alcohol advertising rules was removed entirely; it remained absent from the final version, published after further, minor revisions on 25 May 2016. Specifically, the language around the preferred options for audiovisual commercial communications (AVCCs) changed. The first draft IA sought “to achieve a better balance between competitiveness and consumer protection by, in addition, reinforcing some of the rules seeking to protect the most vulnerable (alcohol advertising exposed to [sic] minors and HFSS foods)”, including through a new obligation “to ensure that minors will not normally hear or see [AVCCs] for alcohol (e.g. via watershed or technical measures)” (p .49). The final published IA, on the other hand, was reframed to “address the lack of level playing field caused by the regulatory burden on providers […] while encouraging the development and improvement of codes of conduct to protect minors from alcohol advertising and from inappropriate AVCCs for HFSS foods” (p. 16).

### 3.1 How did the framing of problems and solutions shift?

The framing of problems and solutions related to AVCCs shifted across IA report versions. The first draft identifies two policy problems: an “uneven playing field” weakening the internal market for audiovisual services, driven by the lighter regulatory regime that exists for on-demand services compared to TV broadcasters, and a deficit of consumer and minor protection, uneven across the region. The revised draft introduces a third problem which remains present in the final IA: the rules on AVCCs are “no longer fit for purpose” (p. 13). In so doing, it reframes AVCCs as an area in need of simplification to support competitiveness.

The first draft explicitly problematises alcohol and HFSS food advertising as a consumer protection problem to be addressed in a revised AVMSD. Minors’ continued exposure to alcohol advertising, in particular, was highlighted as a prominent issue, with an acknowledgement that “the implementation of the related AVMSD provisions by some Member States has raised doubts as to their effectiveness” (p. 7). Conversely, in line with a shift away from problematising the consumer protection aspects of marketing, the revised draft does not discuss alcohol or HFSS advertising. Though reintroduced in the final version, the framing differs markedly in that the stated need for action is restricted to the “improvement” or “strengthening” of self- and co-regulatory initiatives (p. 12).

Overall, our analysis reveals a narrowing of the problem framing from balancing consumer protection and internal market concerns to a positioning of existing rules as an impediment to broadcasters’ competitiveness and requiring “simplification”. The terms “rigid” and “fit for purpose”, for instance, do not appear in the original draft of the IA report but are frequent in later versions. Moreover, specific problematisation of alcohol and HFSS marketing oscillates between a strong health dimension in the first draft and silence in the revised draft, returning in weaker form in the final version.

HFSS and alcohol advertising is also the main area where assessments of existing AVMSD provisions on AVCCs vary: an initial position that existing rules should be “strengthened” shifted to an acceptance of existing rules as largely effective in the final version. All three IA reports are consistent in their diagnosis that rules on sponsorship, product placement, and quantitative limits for broadcast advertising should be more flexible, to enable broadcasters to collect additional advertising revenue and reduce regulatory fragmentation.

Following on from this problem diagnosis, the first draft proposes to introduce a mandatory requirement for member states to “ensure that minors will not normally hear or see” AVCCs for alcohol, including via watershed measures. It notes that “a well-adapted watershed in peak time would prevent minors from being exposed to most of the alcohol advertisements” (p. 51). By contrast, the final version explicitly dismisses watersheds, offering only strengthened self- and co-regulation as a proposal to change the status quo. The first and final version of the IA report both recommend the development and strengthening of codes of conduct for HFSS food advertising, similar to that found in the EU Pledge. Overall, the proposed changes to marketing rules in the first draft aim for a “better balance between improving the competitiveness of TV broadcasters and limiting advertising for consumers” (p. 20), while later versions emphasize simplification and cost savings in this area. The latter is exemplified by changes in the Commission's summary assessment across versions of the IA report, synthesised in Table 1.

**Table 1:** Comparison of policy options for commercial communications across versions of the IA report. The criteria and assessments are reproduced directly from the summary impact tables found on p. 51 of the first IA report draft (February 2016), p. 47 of the revised draft (March 2016), and p. 53 of the final version (May 2016). ‘+’ and ‘-’ signify positive and negative impacts, respectively, as compared to the status quo. The IA reports provide no quantitative criteria or clear definitions for these indicators, or the ‘low’-‘high’ assessments.

Policy options^a^	Internal market impacts	Establish the conditions to ensure competitiveness	Safeguard the protection of minors and consumer protection	Support European cultural diversity	Strengthen access to information and media pluralism	Costs (administrative & compliance)	Effectiveness	Coherence	Feasibility (technical & political)
First draft IA, February 2016									
*More flexible advertising rules (‘sub-options A1/A2’)*	–	+++	–	+++	++	Low	Medium	Medium	Low
*Reinforcing qualitative rules on alcohol while rendering some rules more flexible (‘sub-option B + A1’)	–	++	+	++	+	Medium	High	Medium	Medium
Revised IA draft, March 2016									
*More flexible advertising rules	[n/a]^b^	+++	[n/a]	[n/a]	[n/a]	Low	Medium	Medium	Medium
Final IA, May 2016									
*More flexible advertising rules	[n/a]	+++	[n/a]	[n/a]	[n/a]	Low	Medium	Medium	Medium

a We list all policy options (other than the status quo) assessed in the IA reports. * denotes the option highlighted as the policy preference by DG Connect.

b ^‘^n/a’ (added by the authors) denotes that these impacts were not presented in the summary impact table of the corresponding IA version.

### 3.2 Evidence use in the AVMSD impact assessment

Our analysis of evidence cited in the context of AVCC rules points towards a shift in how existing sources were framed and used, rather than the addition of new evidence during the revision of the IA report. This re-use of sources for drastically different positions is particularly stark throughout two key sections from the first and final IA reports pertaining to the exposure of minors to alcohol advertising and the idea of time-bound advertising restrictions (‘watersheds’), which were proposed in the first draft IA report (Table 2). Despite drawing on the same source – a report by consultancy firm Ecorys and the Finnish National Institute for Health and Welfare for DG Connect, 2015 – the sections reach different conclusions regarding the desirability of alcohol advertising watersheds. Notably, the cited study was focused on minors' exposure to alcohol advertising and did not set out to assess the effectiveness of policy options.

**Table 2:** Comparison of key sections discussing alcohol advertising watersheds and minors’ exposure to alcohol advertising across versions if the AVMSD impact assessment report. The revised draft (dated 25 March 2016) did not discuss alcohol advertising rules. Emphasis added by the authors.

First draft of IA report (February 2016)	Final version of IA report (May 2016)
**“Reinforcing the rules on alcohol advertising would have a positive impact on minors**. In particular, **a well-adapted watershed in peak time would prevent minors from being exposed to most of the alcohol advertisements**. The study on minors' exposure to alcohol advertising showed that minors in most of the 9 selected Member States see most alcohol advertising in TV broadcasts during a certain peak hour. In the Netherlands, where a watershed is in place between 06:00 and 21:00, the average number of impacts for alcohol advertising seen by minors aged 4–14 during peak hour was lower than those in Germany, the UK or the Czech Republic which do not apply watersheds. This approach is supported by consumer organisations from the health sector in their replies to the public consultation. However, **one pitfall of such watersheds may be a shift of alcohol advertising just after peak time**, at a time when minors, although less numerous, are still watching television quite massively.” (p. 51) **“As regards exposure of minors to alcohol advertising, the implementation of the related AVMSD provisions by some Member States has raised doubts as to their effectiveness.** The study to measure minors' exposure to alcohol advertising shows that on TV broadcasting "on average, a minor in the EU saw 200 alcohol impacts and an adult over 450 during one year (2013)". The analysis revealed also that 87% of the television advertisements for alcohol contained at least one element that can be considered to be appealing to minors. […] Although alcohol advertisements are the least recalled type of advertisement by minors aged 9–17 in the nine selected Member States, 23.9% of these minors recalled having seen an alcohol advertisement online in the last month. 63% of the online advertisements (banners) for alcohol contained at least one element that can be considered to be appealing to minors.” (p. 7)	“Member States have been active in this domain in order to protect viewers, and in particular minors, from exposure to alcohol advertising: 24 of them have adopted stricter rules in this area and a number of them have defined the time before which alcohol advertising cannot be broadcast (i.e. watersheds). However, **one major pitfall of such watersheds may be a shift of alcohol advertising just after peak time**, at a time when minors, although less numerous, are still watching television quite massively. As the **study on minors' exposure to alcohol advertising showed, when the time is not well adapted, minors may be exposed quite heavily to alcohol advertising just after the watershed**. Moreover, given the divergences among Member States in peak viewing times for minors, when coupled with the COO principle, watersheds appear less efficient. The applicable watershed would be the one at the country of origin, while minors might be still watching TV in the country of destination.” (p. 12) “The study to measure minors' exposure to alcohol advertising shows that "on average, a minor in the EU saw 200 alcohol impacts and an adult over 450 during one year (2013)". This means that 1.8% of all advertising seen by minors (under age 18) in 2013 was for alcoholic beverages (as compared to 2.2%. for ads seen by adults). In other words, children are exposed to one impact every two days, and at nearly half the rate of adults. […] At the same time, the **majority of countries have self- or co-regulatory schemes in place. Some of them are very efficient, while for others, there is scope for improvement.**” (p. 12)

Overall, patterns of evidence citation across the IA reports indicate that discussion of HFSS food and alcohol advertising, and potential ways to address its impacts, primarily drew on studies commissioned by DG Connect, followed by stakeholder views and external reports, and a smaller number of other source types, such as news articles. Notably, none of the IA reports cite any studies published in peer-reviewed journals in the context of AVCCs.

Studies conducted or commissioned specifically to evaluate and revise the AVMSD form a core part of the cited evidence across IA versions. In addition to the Ecorys et al. (2015) report noted above, this included a study on the impacts of different scenarios for advertising rules (Ramboll Management Consulting, SQW, Visionary Analytics, Institute of European Media Law, 2016), which is cited primarily to support statements on predicted costs, a study on the effectiveness of self and co-regulation (Panteia & Europe, 2016), and the first and second AVMSD implementation reports.

Views and information from consultation with stakeholders, regulators, and government representatives are referenced across all versions of the IA report, primarily to present different constituencies' positions on problems and what should (not) be done about them. Consultation input is used most extensively in the first draft IA report (26 instances); this was halved in the second draft (12 instances) and final IA report (13 instances). Regarding HFSS food and alcohol advertising, the public health community's concerns about the ineffectiveness of existing provisions, and accompanying calls for stronger rules, are referenced in the first draft; this notes, for instance, that “[o]rganisations from the public health sector stress the ineffectiveness of the current provision on HFSS food advertising and the limits of codes of conduct” (p. 45). This acknowledgement does not feature in the second draft, and the final IA report only briefly notes that the public health community supports stronger alcohol rules, positioning this at odds with the view of many broadcasters, food and beverage industry actors, advertisers, and some member states.

Other sources that are cited less frequently include stakeholder publications, news articles, and websites. The first draft refers to WHO publications and the EU's own Action Plan on Childhood Obesity, which problematise childhood obesity in the region. These references do not feature in later versions of the report, though the final IA does contain a link to the High-Level Group on Nutrition and Physical Activity website. Across versions of the IA report, the most cited external publication originates from EGTA, a business association that represents the interests of television and radio sales houses. The report, titled ‘costs and benefits of compliance with the AVMSD’ and not, to our knowledge, publicly available, appears to be the main source for quantitative estimates of revenue increases from more flexible advertising rules.

What is striking across the IA reports (particularly later versions) is the silence surrounding the substantial evidence on the health implications of HFSS and alcohol marketing, and effective ways to mitigate them. This is in spite of several consultation submissions by the public health community which highlight scientific evidence syntheses and individual studies on the health impacts of exposure to alcohol and HFSS advertising, and support advertising restrictions as effective interventions (Anderson et al., 2009; Galbraith-Emami & Lobstein, 2013; Merkur et al., 2013), some of which were EU-funded (De Bruijn, 2012; Science Group of the; European Alcohol and Health Forum, 2009; Winpenny et al., 2012). Notably, several of these sources were reviewed for the Ecorys et al. (2015) report.

### 3.3 The ‘why’: tracing causal pathways underlying the marginalisation of health

In reconstructing the process through which the Commission's proposal for a revised AVMSD was developed, we can explore the range and relative importance of factors that shaped its content. Our process tracing identifies and assesses three factors which may have contributed to the de-prioritisation of health concerns during the development of the Commission proposal: a shift in the evidence base, the RSB review process, and stakeholder pressure.

Written documentation shows that, when prompted, DG Connect justified the ruling-out of tighter advertising rules with reference to a lack of evidence of effectiveness. Responding to a query by the health directorate (DG SANTE), DG Connect argued that “restrictive measures such as watersheds have not proven very useful and could lead to the unwanted effect of concentrating alcohol advertising around certain times of the day, when many children may still be in front of the screen”, noting that “it is considered more effective to strengthen self- and co-regulatory frameworks in this area, as well as for [HFSS] food” (DG Secretariat-General, 2016a). Yet, our analysis of citations across the three versions of the IA report, presented above, identifies no new sources which could reasonably explain this assessment and DG Connect's shift in position, instead showing an inconsistent utilisation of a report on minors' exposure to alcohol advertising across European countries (Ecorys et al., 2015) to justify opposing claims on advertising watersheds. Since, under the Better Regulation guidelines, one would expect any new, decisive evidence or analysis to be cited, this suggests that the decision to de-prioritise health was driven by other factors.

DG Connect officials were required to revise the rejected IA report in light of the RSB's suggestions. None of the written recommendations, however, explicitly address the proposal for tightening advertising rules. Rather, they make broader calls to “present a more focused, evidence-based and sufficiently clear and concise analysis”, clarify the context and scope of the initiative, “revise the problem statement and back it up with appropriate evidence, simplify the presentation of options and their impacts”, and “emphasize the simplification/burden reduction elements of the set of preferred options”. A later comment by the RSB on the revised IA draft notes that the “streamlined problem description left behind some of the issues identified in the evaluation”, including “consumer protection issues linked to the advertising of HFSS foods and alcohol” (p. 2). This suggests some surprise at the side-lining of health and likely explains the re-integration of some health-focused language in the final IA report. DG Connect's written response to this RSB comment, appended to the final IA, stated that “there is not sufficient evidence available to warrant the need to go further” than a re-enforcement of self- and co-regulation (p. 62).

As such, written documentation does not indicate that the RSB explicitly encouraged the de-prioritisation of health. However, the possibility for DG Connect officials to have interpreted the Board's feedback – perhaps most notably that reinforcing the Commission's stated aims of simplification and burden reduction – as requiring a shift away from additional rules, including on HFSS food and alcohol, remains. Similarly, the preference for self- and co-regulation aligns with efforts by the Commission to ensure such non-regulatory measures are always considered when developing policy (European Commission, 2015a, p. 23). Though, in this case, we were not able to collect the necessary interview data to evaluate the role of these wider norms, this highlights the important of exploring the individual-level logics behind observable changes.

The Commission's dismissal of strengthened restrictions on harmful marketing further aligns with long-standing opposition to such approaches by commercial actors from the advertising, food and beverage, and broadcasting industries. These positions were voiced throughout the consultation process which informed the IA and the wider AVMSD revision. Major alcohol producer Bacardi-Martini BV, for instance, argued in its submission that “instead of strengthening the Directive, endorsing regulatory bodies and enforcing the Directive will be more effective in maintaining the high standards of advertising and marketing across the industry” (p. 7) – a position mirrored in the final legislation. Official meeting declarations suggest that DG Connect and other relevant DGs engaged consistently and repeatedly with commercial actors during the agenda-setting stage, including on marketing rules. Relevant officials discussed the AVMSD in meetings with several broadcasting industry groups – including Sky Group, News Corporation, and the European Broadcasting Union – between the submissions of the first and the second IA report draft. Alcohol producer Heineken discussed an "initiative on alcohol advertising" with cabinet members of the Commissioner for Digital Economy and Society in early February (Heineken, 2023). While it remains possible that interest group pressure (including from the broadcasting industry, which generally did not support stronger rules on food or alcohol advertising) contributed to the final outcome, limited available evidence prevents us from ascertaining a direct link.

## 4 Discussion

Throughout the IA process that informed the European Commission's 2016 proposal for a revised audiovisual media services directive (AVMSD), we observe a shift from policy recommendations that included strengthening the rules on alcohol advertising, to a recommendation to promote and improve voluntary codes of conduct. This represents only a marginal change from the status quo, inconsistent with the public health evidence base. For food high in fat, sugar, and salt (HFSS), there is no evidence that substantially strengthened rules were considered. Notably, the original policy proposal for advertising rules was not formally discarded, which would require justification within the IA report, but rather disappears, leaving only the more flexible rules and the status quo as available options. Though the revised Directive retains an overarching objective to enhance consumer protection, the relevance of marketing rules to this goal, and specifically the health rationale for strengthening protection, is diluted. In essence, the stronger rules were rejected by positioning more flexible rules (vis-à-vis the status quo) as the only available option.

On the advertising of alcohol and HFSS foods specifically, this dilution of the health dimension corresponds to a shift in framing and inconsistency in the use of evidence. Recognition of the weaknesses of the existing rules evolves, between the first and final IA reports, into a discussion of how they can be improved without a change in governance approach. This is accompanied by a shift towards a focus on regulatory simplification and the competitiveness of the broadcasting sector as a whole, exemplified, for instance, by the terms “rigid” and “fit for purpose”, which are only introduced in later iterations. Bizarrely, the key arguments against tighter restrictions (in the form of watersheds) in the final version draw on the same study previously invoked to support the opposite position. Furthermore, our analysis finds no indication within the IA reports that the (by then substantial) body of peer-reviewed evidence on the effectiveness of different approaches to minimising children's exposure to advertising for unhealthy commodities was comprehensively considered by DG Connect. This is despite the inclusion of and reference to relevant research within submissions to the consultation process (DG Connect, 2015). The Better Regulation guidance in place at the time sets out how indirect health impacts should be identified, and requires consideration of whether these impacts affect specific populations (explicitly noting children as an example risk group) (European Commission, 2015a, 2015b). Our analysis, however, indicates that the health impacts of (in)action were not systematically assessed in this case, and that some of the evidence considered and funded by the Commission was absent from the final published IA report.

This speaks to wider debates on the politics of ‘evidence-based policymaking’. As Parkhurst (2017, p. 6) argues, “rather than being apolitical, the appeal to evidence, or to particular forms of evidence, can be decidedly political by promoting a de facto choice amongst competing values.” Our findings point towards what Parkhurst terms *technical bias,* which refers to the strategic use and omission of evidence in policymaking. It has already been noted that Better Regulation may foster technical bias and that “the dominant meaning of evidence [that Better Regulation embodies] lends itself well to politicization” (Godziewski, 2020, p. 1314). The agenda enshrines IA processes that favour a particular type of (quantitative) evidence, and require (in)action to be justified with reference to (the lack of) this evidence, even where such decisions may be politically determined. As such, acknowledging the strategic, political value of evidence – as specific sets of knowledge and as a more abstract idea – is crucial, particularly in the context of outwardly technical, ‘evidence-based’ decision-making.

This research is subject to several limitations. The available data are restricted by incomplete proactive transparency – for instance, only meetings with senior officials are declared and meeting minutes are not routinely published – and “systemic delays in processing public access to documents requests” (European Ombudsman, 2023). Notably, we do not currently have sufficient evidence to assess the presence or relevance of pressure from elsewhere in the Commission as an additional contributing factor, though we know from internal documentation that the issue was discussed "at the political level" DG Secretariat-General (2016b). Fast-track interservice consultation meeting, held on 22 April 2016 (source: access-to-document request EASE 2022/6217). Whilst access-to-document requests have been used to reveal internal pressures and politics in, for instance, the development of tobacco control policy (Peeters et al., 2016), at the time of submission, some of our own requests remain unaddressed, pending decisions on confirmatory applications (see Appendix B). Further, whilst interviews with officials would provide first-hand insights into the mechanisms which led to the dismissal of stronger advertising rules, our requests to relevant officers were denied or unanswered.

### 4.1 Conclusions

Our analysis demonstrates the importance of examining the black box of EU agenda-setting. Whilst the final law is adopted in the legislative stage, the proposal underlying it – and in which the policy options under discussion are decided – is developed far earlier. In line with calls for public health research to engage with the political sciences (Gómez et al., 2022; Greer et al., 2018), we focus attention on the institutions of agenda-setting and their role in determining the scope and parameters of health policies. Whilst some of these internal dynamics – particularly those between different parts of the Commission – remain ‘black-boxed’, our findings offer novel insights by identifying the exact point at which stronger rules for public health were ‘taken off the table’. To some extent, our observations on the role of the RSB speak to reservations voiced by civil society groups regarding the dampening effect that this ‘quality control’ mechanism may exert on health and environmental protections (Brooks and Lauber forthcoming; Corporate Europe Observatory, 2022; New Economics Foundation & European Environmental Bureau, 2020). While we find no evidence of direct RSB pressure to lower regulatory ambition, changes made throughout the IA review process align with the Board's overarching push towards burden reduction and simplification (in line with wider Better Regulation guidance). Further, in-depth research is required to unpack the role of institutions such as the RSB, and overarching norms like simplification and burden reduction, which are engrained in the Better Regulation agenda and inform decision-making across sectors.

Public health organisations are calling for the regulation of unhealthy food marketing at EU level, through a separate Directive, led by DG SANTE rather than DG Connect (European Public Health Alliance, 2021). Although the case for comprehensive, mandatory rules on food marketing has only grown stronger since the revision of the AVMSD (Boyland et al., 2022) our findings indicate that this is no guarantee that the IA process will adequately reflect this. In seeking to effectively regulate the commercial drivers of ill-health, it is important to consider the processes, actors, and policy dynamics that shape how rules are made.

## Funding

This work was supported by 10.13039/100014013UK Research and Innovation [10.13039/501100000265Medical Research Council grant reference MR/T023244/1]. The funder had no role in study design, data collection, analysis, decision to publish, or preparation of the manuscript. For the purpose of open access, the author has applied a Creative Commons Attribution (CC BY) licence to any Author Accepted Manuscript version arising from this submission.

## Ethics statement

This research does not directly involve human participants. The wider project has received ethical approval from the University of Edinburgh School of Social and Political Science [ID 284499].

## Declaration of competing interest

The authors declare the following financial interests/personal relationships which may be considered as potential competing interests: Eleanor Brooks is a Scientific Advisor to the European Public Health Alliance (EPHA), and the EPHA is a partner on this UKRI-funded project. EPHA had no role in study design, data collection, analysis, decision to publish, or preparation of the manuscript.

## Figures and Tables

**Table:** 

EASE number	Date requested	Summary of requested documents	Response date	Summary of response
2022/2304	22 April 2022	1st version of IA report	12 May 2022	Received in full.
2022/3715	28 Jun 2022	Documentation of interactions with Regulatory Scrutiny Board, inter-service steering group, and external stakeholders regarding AVMSD revision, 2nd version of IA report.	21 Sept 2022	Partial response; unaddressed elements rolled into new request 2022/5491.
2022/5491	21 Sept 2022	2nd version of IA report, documentation of interactions with external stakeholders and DG Secretariat-General regarding AVMSD revision.	11 Nov 2022	Partial response; authors submitted confirmatory application, challenging response, on 21 Nov 2022. Awaiting response at time of writing - see below regarding ongoing European Ombudsman complaint.
2022/6217	28 Oct 2022	Documents relating to internal deliberations within DG Connect relating to the AVMSD revision.	20 Jan 2023	Partial response; authors submitted confirmatory application, challenging response, on 26 Jan 2023.Prompted by European Ombudsman in October 2023 following author complaint about handling of (EASE 2022/5491 & 2022/6217) but DG Connect has not complied with response deadlines.

**Table:** 

Document(s)	Source	Pages
Inception Impact Assessment for REFIT evaluation and impact assessment of the EU AVMSD (Oct 2015)	European Commission website	13
Contributions and preliminary trends of the public consultation on Audiovisual Media Services (Oct 2015)	European Commission website	3
Ex-post REFIT evaluation of the AVMSD (May 2016)	EUR-Lex	58 (241 with annexes)
First draft of the impact assessment report for the revision of the AVMSD (Feb 2016)	Access-to-document request GestDem 2022/3715	77
Second draft of the impact assessment report for the revision of the AVMSD (Mar 2016)	Access-to-document request GestDem 2022/5491	51
Impact assessment report for a revised AVMSD (May 2016)	EUR-Lex	58 (358 with annexes)
Proposal for a revised AVMSD (May 2016)	EUR-Lex	33
RSB opinion on the impact assessment report for the revision of the AVMSD (first draft, version of 22 February 2016) (Mar 2016)	Access-to-document request GestDem 2022/2304	3
RSB opinion on the impact assessment report for the revision of the AVMSD (resubmitted version of 25 March 2016) (April 2016)	European Commission website	3
Meeting declarations	Integrity Watch (uses Commission data)	n/a

*Hyperlinks are provided where the documentation is publicly available.

**Table:** 

IA version, page(s)	Item(s) of evidence cited	Source type	Source	Topic(s)^1^	Context (direct quotes of all relevant text citing evidence in food/alcohol advertising context; footnotes in brackets, [REF] indicates reference placement)
v1, p. 7	Study on the exposure of minors to alcohol advertising	Commissioned study	Ecorys et al. (for DG Connect)	Alc	As regards exposure of minors to alcohol advertising, the implementation of the related AVMSD provisions by some Member States has raised doubts as to their effectiveness. The study to measure minors' exposure to alcohol advertising [REF] shows that on TV broadcasting "on average, a minor in the EU saw 200 alcohol impacts and an adult over 450 during one year (2013)". The analysis revealed also that 87% of the television advertisements for alcohol contained at least one element that can be considered to be appealing to minors.
v1, p. 7	OPC response: Irish Heart Foundation [example]	Stakeholder input	Consultation	AVCC-gen	In particular, as regards minors, some consumer organisations [REF] claim that their ability to recognise adverts on websites is below their ability to recognise adverts on TV. This could make them more vulnerable to digital marketing.
v1, p. 7	Study on the exposure of minors to alcohol advertising	Commissioned study	Ecorys et al. (for DG Connect)	Alc	As regards alcohol online advertising, out of 1319 screen captures for YouTube, only two unique prerolls for alcohol brands were captured. One of the pre-rolls advertised a non-alcoholic drink and was only served on the profile of an adult. The other pre-roll was captured three times for a minor's profile and three times for an adult's profile *[However, this could be underestimated because of several caveats in the methodology: For example, cookies were generated during two months of online activity, whereas minors will typically have a richer browser history than the profiles that were generated for the purpose of this study. In addition, the data capture had a limited focus and only generated activity on a computer, whereas minors typically use different devices to go online. Also, there was no spill-over between profiles, whereas in reality minors may share a device with adults]*. Yet at the same time, when surveyed, minors perceived to have been exposed quite substantially to alcohol advertising online. Although alcohol advertisements are the least recalled type of advertisement by minors aged 9–17 in the nine selected Member States, 23.9% of these minors recalled having seen an alcohol advertisement online in the last month *[This could be overestimated because of other caveats in the methodology: First, the survey relies on self-reporting measures of exposure by asking recall questions, which induces the risk of recall-bias. Hence, where minors indicated that they recall having seen (alcohol) advertisements, it does not necessarily mean that the (alcohol) advertisement was actually provided. Second, no strict definition of advertisement was provided in the survey and therefore the reported exposure to alcohol advertising is subject to interpretation of what is perceived as ‘alcohol advertising’ by minors. For example, minors may have indicated that they recalled seeing an advertisement, whereas in reality, they saw user-generated content rather than commercial communications produced by advertisers]*. 63% of the online advertisements (banners) for alcohol contained at least one element that can be considered to be appealing to minors.
v1, p. 7	1st AVMSD Application Report (2012)	EU policy document	European Commission	HFSS	As regards commercial communications for HFSS foods, an evaluation of the Platform for Action on Diet, Physical Activity and Health concluded that stakeholders' initiatives in the field of marketing and advertising showed good progress. However, their impact could be further strengthened [REF].
v1, p. 7	[REF1] WHO European Childhood Obesity Surveillance Initiative, Implementation of round 1 (2007/2008) & round 2 (2009/2010)	External report	WHO Europe	HFSS	According to estimates from the WHO's Childhood Obesity Surveillance Initiative (COSI), around 1 in 3 children in the EU aged 6–9 years old were overweight or obese in 2010. This is a worrying increase compared to 2008, when estimates were 1 in 4 [REF1+2].
	[REF2] EU Action Plan on Childhood Obesity 2014–2020	EU policy document	European Commission	HFSS	
v1, p. 7-8	[REF1] EU Action Plan on Childhood Obesity 2014–2020	EU policy document	European Commission	HFSS	As underlined in the 2014–2020 EU Action Plan on Childhood Obesity, this situation derives from a number of behavioral risk factors, including minors' exposure to food advertisements and other marketing tactics on TV [REF1+2].
	[REF2] WHO Regional Office for Europe, Marketing of foods Action high in Plan fat, salt on and Childhood sugar to Obesity children: update 2012–2013	External report	WHO Europe	HFSS	
v1, p. 8	CNN, Kids seeing more unhealthy snack ads, report says (Nov 2015)	News media	CNN	HFSS	In addition, some online advertising for foods high in fat, salt and sugar (HFSS) has been identified around certain online videos that attract minors' attention [REF].
v1, p. 8	EAO, Online Advertising in the EU, 2015.	Commissioned study	European Audiovisual Observatory	AVCC-gen	In contrast, the total size of the online advertising market in the EU in 2013 has increased by 11.6% compared to 2012. Online is the second medium in Europe for ad spend, just behind TV advertising, though it surpassed TV advertising in 2014 in a number of Member States [REF] *[this varies widely across Member States: online ad spend per capita ranges from €138 in the UK to €2 in Romania, while the top three countries for online ad spend in the EU in 2014 (UK, Germany and France) accounted for 66.7% of online advertising in Europe].*
v1, p. 9	UK Digital Upfronts 2015, Enders Analysis [paywalled]	External report	Enders Analysis	AVCC-gen	According to the European Audiovisual Observatory, online video advertising revenues are expected to grow from EUR 1.9 billion to EUR 4.1 billion by 2018 with a market share of almost 60 % for Facebook and YouTube. This is particularly relevant where these services more directly compete for the same advertising market as TV broadcasters, especially when it comes to attract the attention of millennials *[according to* [REF]*, "YouTube again emphasised its reach among 16-34s at a time when TV viewing among millennials is in steady decline. (…) This was at the heart of Google's pitch to brands last, but there was a more specific pitch this time [:] (…) brand advertisers seeking to reach 16-34s should move 24% of TV ad budgets to YouTube to optimize reach and efficiency"].*
v1, p. 9	[REF1] Etude CNC l’économie de la télévision de rattrapage en 2014	External report	Centre National du Cinema et de L'Image Animee	AVCC-gen	In the online market where TV broadcasters are currently mostly present, i.e. the on-demand services covered by the AVMSD, the share of advertising revenue remains modest. For example, free-to-view UK online TV services such as ITV Player and All 4 generated just GBP 240 million in advertising in 2014, equivalent to 5.6% of the total TV advertising and sponsorship market in the UK. In France, the revenues from advertising on catch-up TV services amounted to EUR 80 million in 2014 [REF1] which represents 0.7% of the revenues generated by French TV broadcasters in 2013 [REF2].
	[REF2] EAO REFIT data Note B.1: market revenues and investments – linear services	Commissioned study	European Audiovisual Observatory	AVCC-gen	
v1, p. 15	Study on the exposure of minors to alcohol advertising	Commissioned study	Ecorys et al. (for DG Connect)	Alc	As regards specifically alcohol advertising, the study on minors' exposure to alcohol advertising revealed a great number of measures put in place by the industry to prevent exposure of minors to alcohol advertising.
v1, p. 28	European Advertising Standards Alliance	Stakeholder input	Consultation (presumed)	AVCC-gen	For HFSS advertising, the self-regulation organisations' secretariats budget currently range from small (with just one to five members of staff and a budget up to EUR 250 000) to large (up to over 100 members of staff with budgets up to and over EUR 1 000 000) and cover the whole advertising field. SROs' secretariats mainly receive the complaints, gather any necessary information about the complainant and evidence of the advertiser in order to prepare the case for jury. These SROs are either funded by membership fees (18 of them) or a levy system (5) from the industry [REF].
v1, p. 28	OPC response: EURALVA	Stakeholder input	Consultation	AVCC-gen	As regards commercial communications, consumer organisations underline that minors should be equally protected in the traditional linear environment and in online media and that provisions should equally apply to all platforms and programs [REF].
v1, p. 29	OPC response: BEUC	Stakeholder input	Consultation	AVCC-gen	For some, it does not mean that the rules on commercial communications necessarily need to be the same [REF].
v1, p. 29	OPC responses: Alcohol Focus Scotland, Alcohol Health Alliance, European Alcohol Policy Alliance, UK Institute of Alcohol Studies, IOGT International, Nordic Alcohol and Drug Policy Network, VAD [examples]	Stakeholder input	Consultation	Alc	In addition, several stakeholders [REF] from the public health sector stress that provisions on audiovisual commercial communications for alcoholic beverages should also be extended to online services.
v1, p. 29	OPC responses: World Cancer Research Fund, World Obesity Federation, European Association for the Study of the Liver [examples]	Stakeholder input	Consultation	HFSS	Health organisations also support a regulation of all forms of food and beverage marketing and advertising, including online platforms and providers hosting user-generated content [REF].
v1, p. 29	OPC responses: EBU, CIRCOM, France Télévisions [examples]	Stakeholder input	Consultation	AVCC-gen	Among public service broadcasters, some [REF] favour an extension of the rules on AVCCs to all electronic commercial communications.
v1, p. 29	OPC responses: DE, SI, IT, AGCOM [examples]	Stakeholder input; Member state	Consultation	AVCC-gen	A few regulators and Member States raised in the public consultation [REF] the possibility to extend the minimum set of qualitative rules to all forms of commercial communications on the Internet.
v1, p. 29	OPC responses: IE, ES CNMC [examples]	Stakeholder input; Member state	Consultation	Alc/HFSS	Some mentioned this for alcohol and HFSS foods advertising rules [REF].
v1, p. 29	OPC responses: DE, FR, AT, BE-Fr, IT, LV, PT [examples]	Member state	Consultation	AVCC-gen	Several Member States [REF] called for an extension of the scope of the AVMSD to new online services, in particular for the rules on public health.
v1, p. 43	SQW/Ramboll study	Commissioned study	SQW & Ramboll (for DG Connect)	AVCC-gen	According to regulators, the existing requirements have resulted in administrative costs up to EUR 1.235 million per year per regulatory authority [REF] *[depending on the size of the audiovisual market in the country, the staff costs, the use of external services for monitoring].*
v1, p. 44	European Advertising Standards Alliance	Stakeholder input	Consultation (presumed)	HFSS/Alc	In the majority of Member States, self- and co-regulation systems are in place in the field of advertising in general and frequently on HFSS foods and alcohol advertising. These systems are either funded by membership fees or a levy system from the industry and their cost ranges from EUR 250 000 to EUR 1 000 000 [REF].
v1, p. 44	OPC responses: advertisers, broadcasters, MS, regulators	Stakeholder input	Consultation	AVCC-gen	In their replies to the public consultation, advertisers, some broadcasters and several Member States and regulators claim there is no level playing field between TV broadcasters and other media services, and in particular between TV broadcasters and on-demand service providers. However, a few broadcasters, mainly from the UK, prefer the status quo option.
v1, p. 44	Survey response: Ofcom	Stakeholder input	Consultation	PPlacement	Moreover, due to the restrictive character of the rules on product placement, this advertising format has not delivered its full potential in terms of revenues. Some regulators and Member States confirm that the rules create legal uncertainty for stakeholders, discouraging them to invest in product placement. As a benchmark, in the US, where there is no material regulation of product placement, this format represents almost 5% of the TV ad spend market. In the UK, it represents a share of only 0.1% [*in the US, TV ad spend for 2014 was $69.4 billion with a mid-level forecast of $3.5 billion for product placement. In the UK, the ad market for 2014 was worth £3.56 billion, with product placement capturing £3.5 million of this market*] [REF].
v1, p. 44	1st AVMSD application report	EU policy document	European Commission	AVCC-quant	As regards the hourly limitation, most broadcasters consider that the lack of flexibility of the 12 min rule and the restrictive character of its exceptions prevent them from maximising their revenues around peak periods. The monitoring of advertising in Member States has indeed shown that this rule is regularly breached in a number of Member States [REF1+2].
	2nd AVMSD application report	EU policy document	European Commission	AVCC-quant	
v1, p. 44	[UNCLEAR] large commercial broadcaster, EGTA	Other	Unclear	AVCC-quant	According to a large commercial broadcaster, further liberalisation of insertion rules holds potential additional revenue of up to 6–8% (rough estimate). Similarly most EGTA members estimated that revenues could increase from between 1% and 10% if the current rules were to be relaxed without providing more details.
v1, p. 45	OPC response: BEUC, EURALVA	Stakeholder	Consultation	AVCC-gen/AVCC-quant	Consumer organisations (including those from the health sector) recognise the relevance of the rules but think that they are neither fair nor effective. In particular, consumer organisations underlined the need not to lower consumer protection [REF] *[In addition EURALVA underlines that the quantitative rules are not satisfactory if not respected by stakeholders]*.
v1, p. 45	Ofcom's report on UK audience attitudes to the broadcast media 2014	External report	Ofcom	AVCC-quant/Alc	While consumers still have some concerns about excessive advertising on TV [REF] *[in 2014, for example, 57% of UK viewers agreed with the statement “there are already more minutes of advertising in an hour than I am really happy with”. However, viewers also appear to understand the relationship which exists between advertising and the funding of content: 72% of UK viewers questioned in 2014 identified without prompting that advertising represented the primary source of funding for the UK's three main free-to-air commercial television services (ITV/STV/UTV, Channel 4 and Channel 5) which between them account for 24% of UK adult television viewing and just under £1.5bn (€2.1bn) in programme spend]*, one of their main concerns relates to alcohol.
v1, p. 45	Study on the exposure of minors to alcohol advertising	Commissioned study	Ecorys et al. (for DG Connect)	Alc	A study on minors' exposure to alcohol advertising [REF] shows that on average, in 2013, a child in the EU saw 200 alcohol impacts *[impact is a measure of how often a spot is viewed: it yields the absolute number of times a spot was seen over a given timeframe]* and an adult over 450 on television.
v1, p. 45	OPC responses: alcohol NGOs	Stakeholder input	Consultation	Alc	In particular, in their replies to the public consultation, the alcohol associations deem that the AVMSD should restrict the volume of alcohol advertisements rather than their content.
v1, p. 45	OPC responses: public health/civil society	Stakeholder input	Consultation	HFSS	Consumer organisations consider that self- and co-regulation systems take too long to review complaints while advertising campaigns are fast-paced. Organisations from the public health sector stress the ineffectiveness of the current provision on HFSS food advertising and the limits of codes of conduct.
v1, p. 46	SQW/Ramboll study	Commissioned study	SQW & Ramboll (for DG Connect)	AVCC-gen	The incremental costs for TV broadcasting and on-demand service providers of the new provisions [Option A] would be zero *[advertising scheduling is a core component of broadcast programming and the quantitative rules imposed by the AVMSD are only a small part of a large number of parameters taken into account in TV scheduling strategies aiming at optimising audience and revenue. The costs associated with broadcast programming, including IT costs, are “business as usual”, i.e. costs endured even in the absence of the AVMSD]* [REF].
v1, p. 46	SQW/Ramboll study	Commissioned study	SQW & Ramboll (for DG Connect)	AVCC-gen/AVCC-quant	There would not be any incremental administrative costs for regulators and it is likely that they could lower their costs due to a lower level of requirement. In the extreme scenario of a complete removal of the Directive's quantitative rules and of regulators no longer performing monitoring and enforcement activities, current regulatory costs amounting to up to EUR 1 032 985 could be saved on an annual basis *[based on the current average value for the monitoring of 1 linear provider established in the EU (PPP adjusted) which is derived from a sample of the regulatory costs in 7 MS which can be considered as a representative sample of different approaches to fulfilling regulatory responsibilities with regard to the monitoring and enforcement of the quantitative rules. It is further assumed that regulators focus their regulatory activities on linear services which have more than 0,5 % of the audience share]* [REF].
v1, p. 46	SQW/Ramboll study	Commissioned study	SQW & Ramboll (for DG Connect)	AVCC-quant/PPlacement/Sponsorship	However, the likelihood of this assumption materializing is quite low and while removing the 12-min rule has some potential for decreasing monitoring/enforcement costs, the impact would be offset by the need to maintain monitoring activities for the rest of the quantitative and qualitative rules. Similarly, in the extreme scenario of completely removing product placement and sponsorship rules, this could generate respectively up to EUR 2 250 897 and EUR 2 138 780 savings per year at EU level [REF].
v1, p. 46	EGTA's report on the costs and benefits of compliance with the AVMSD	External report	EGTA	AVCC-quant	Most broadcasters consider that the insertion rules are no longer relevant nor are they effective. Some argue that because of these rules, schedules are not built around viewers' comfort or advertisers' demand, which is counter-productive. It is considered that revenues could increase between 1 and 10 % [REF].
v1, p. 46	SQW/Ramboll study	Commissioned study	SQW & Ramboll (for DG Connect)	AVCC-quant	In case of the extreme option of abolishing interruption rules, the overall potential revenue gains could amount to 1,35% of advertising revenues coming from cinematographic works and news programmes. This is however a conservative estimate since the parameters for calculation do not take into account different target groups, time slots etc [REF].
v1, p. 47	[REF1] EGTA's report on the costs and benefits of compliance with the AVMSD	External report	EGTA	PPlacement	Most broadcasters agree that product placement and sponsorship rules should be clarified and simplified. A simplified set of rules on product placement could result in an increase of product placement revenues by approximately 10%–15% [REF1], or a 4% increase in total advertising revenues in the EU (i.e. potentially additional revenue of EUR 1 176 600) [REF2] *[based on the assumption that current revenues on product placement in Europe capture around 0.1% of total ad revenues and only in some cases might go above 1%, while in countries such as US where the regulations on product placement are very loose or virtually non-existing at all, the market share captured by product placement is around 5%. Such significant direct increase can however hardly be expected in reality (at least short to medium time perspective), mainly due to inherent differences between the EU and the US markets]*.
	[REF2] SQW/Ramboll study	Commissioned study	SQW & Ramboll (for DG Connect)	PPlacement	
v1, p. 47	SQW/Ramboll study	Commissioned study	SQW & Ramboll (for DG Connect)	Sponsorship	Allowing more flexibility in sponsorship rules would allow broadcasters to generate additional sponsorship revenues; EGTA members' estimates range from 15% to 50% in sponsorship revenues. This could result in more than EUR 441m increase of total TV advertising spend in the EU (i.e. around 1,5% of current total TV advertising market value) [REF] *[based on an average assumption that of a given range (30%) of an expected increase in revenues from sponsorship activities, as compared to the current estimation that sponsorship captures around 5% (net value) of total TV revenues in their national markets]*.
v1, p. 47	[REF1] EGTA's report on the costs and benefits of compliance with the AVMSD	External report	EGTA	AVCC-quant	A shift to a daily limit could generate between a 2% and 15% increase of revenues [REF1+2] *[EGTA's report on the costs and benefits of compliance with the Audiovisual Media Services Directive [non-confidential version], confirmed by a broadcaster from a small Member State who replied to the public consultation (MTV Oy), this could mean "an increase of advertising income of about 2 million euro annually"].*
	[REF2] OPC response: MTV Oy	Stakeholder input	Consultation	AVCC-quant	
v1, p. 47	TF1/GRILLE DES ÉCRANS	Web	TF1	AVCC-gen	For example, over the last six months of 2015 [REF], the gross price of a 30-s advertising spot during prime time on TF1 was EUR 67 330.
v1, p. 47	Nielsen's Advertising and audiences: State of the media Report, 2014	External report	Nielsen	AVCC-quant	It is estimated that during important events or programmes, European broadcasters could increase their advertising pressure by a few minutes, from 12 min to 14–15 min *[based on the average minutes of commercials aired* per *hour in the USA* [REF]*]*, taking into account European viewers' lesser propensity to stand long advertising breaks.
v1, p. 47	COMPTE DE RESULTAT SOCIAL (Normes Françaises)	External data	TF1	AVCC-quant	This would mean an increase of around 2,5 min of advertising, i.e. 5 more advertising spots of 30 s, which, all things being equal, could translate for a channel such as TF1 in an increased revenue of 336 650 EUR i.e. theoretically EUR 122 million annually i.e. 10 % of the turnover of the channel in 2014 [REF].
v1, p. 48	Ofcom, An econometric analysis of the TV advertising market, 2011	External report	Ofcom	AVCC-quant	Indeed, the demand for advertising is inelastic and an increase in the supply of commercial impacts will be likely to lead to a fall in the prices. However, according to an OFCOM study "the elasticity is significantly higher in peak times, perhaps reflecting the greater choice of high-quality alternative programming that is available during these times" [REF] *[according to an Ofcom study: "a 1% increase in advertising minutage on ITV1 at peak times would result in a 0.002% decrease in viewing demand for programming on that channel"]*. This would mean that a potential fall in the prices would be limited for advertising in peak times and placing advertising spots at that moment of the day could maximize TV broadcasters' revenues.
v1, p. 48	OPC responses: broadcasters & MS	Stakeholder input	Consultation	AVCC-quant	Most commercial broadcasters advocate more flexible advertising rules. However, a few broadcasters (mainly from the UK) deem that it should remain in its current form to keep the advertising market stable. They are supported by some Member States (UK, FR).
v1, p. 48	OPC responses: PC, EMMA, ENPA, BDZV VDZ, MLE, NMA, VOEZ and ANSO [examples]	Stakeholder input	Consultation	AVCC-quant	The printed press industry claims that more opportunities to advertise on TV could imply changes in advertisers' media mix, which may be to their detriment [REF].
v1, p. 48	OPC responses: SACD, VS, SAA, VdFS [examples]	Stakeholder input	Consultation	AVCC-quant	Due to a relaxation of the interruption rules, there could be more frequent and longer advertising breaks. This may thus affect the integrity of cinematographic works. Several right holders' associations have underlined this as a very important point to be safeguarded in their contributions to the public consultation [REF].
v1, p. 48	[REF1] Etats-Unis: et maintenant, moins de coupures de publicité (Feb 2016)	News media	Télérama	AVCC-quant	However, viewers' recent tendency to turn to other offers is leading broadcasters to decrease the amount of advertising on their channels [REF1+2 + 3]. Broadcasters indeed argue that they will not massively increase advertising pressure in order not to drive viewers away.
	[REF2] In Dramatic Overhaul, TNT to Cut Ad Load by 50% (Feb 2016)	News media	Adweek	AVCC-quant	
	[REF3] Viacom To Cut Back On Primetime TV Ads Starting In October (Exclusive) (Sept 2015)	News media	Variety	AVCC-quant	
	Broadcasters (presumably consultation)	Stakeholder input	Consultation	AVCC-quant	
v1, p. 49	SQW/Ramboll study	Commissioned study	SQW & Ramboll (for DG Connect)	Alc	Still as regards alcohol advertising, compliance costs for TV broadcasters would not be significant. Stricter rules on alcohol advertising are already in place in the majority of Member States and thus many broadcasters are already using watersheds for the protection of minors (see ANNEX 16). These mechanisms are in themselves easy to implement and would not generate any additional compliance costs beyond "business as usual" costs of programming advertising [REF].
v1, p. 50	"[Reference to source needed]"	n/a	Unclear	HFSS	For HFSS foods advertising, codes of conduct are already in place in all Member States except two. Developing codes at Union level would imply limited additional costs as they would be mostly absorbed by the current existing structures *[for information, self-regulation organisations' secretariats budget currently range from small (with just one to five members of staff and a budget up to EUR 250 000) to large (up to over 100 members of staff with budgets up to and over EUR 1 000 000) and cover the whole advertising field. SROs' secretariats mainly receive the complaints, gather any necessary information about the complainant and evidence of the advertiser in order to prepare the case for jury. These SROs are either funded by membership fees (18 of them) or a levy system (5) from the industry]* [REF].
v1, p. 50	Study on the exposure of minors to alcohol advertising	Commissioned study	Ecorys et al. (for DG Connect)	Alc	Imposing measures to prevent minors from normally watching or hearing audiovisual commercial communications for alcohol may decrease broadcasters' revenues. In 2013, alcohol advertising spots in the EU (23 Member States) represented on average around 1,9% of the total number of advertising spots (ranging from 4,8% in Croatia to 0,4% in Slovenia) [REF].
v1, p. 50	SQW/Ramboll study	Commissioned study	SQW & Ramboll (for DG Connect)	Alc	It is estimated that alcohol advertising generates about EUR 408 million in yearly advertising revenues for EU TV broadcasters [REF]. A watershed on alcohol advertising would certainly reduce these revenues, especially in the short term.
v1, p. 50	[REF1] SQW/Ramboll study	Commissioned study	SQW & Ramboll (for DG Connect)	Alc	However, even in Member States where there is a complete ban on alcohol advertising on TV (FR, SE), the overall advertising revenues do not seem to be significantly lower than in other Member States. This implies that, in the long run, a new equilibrium will form [REF1+2] and that the time slots used by alcohol advertising may be filled by other advertising.
	[REF2] Study on the exposure of minors to alcohol advertising	Commissioned study	Ecorys et al. (for DG Connect)	Alc	
v1, p. 50	SQW/Ramboll study	Commissioned study	SQW & Ramboll (for DG Connect)	HFSS	As regards HFSS food advertising, it is estimated that it generates about EUR 1342,5 million in yearly advertising revenues for EU TV broadcasters *[the SQW/Ramboll study indicates that the share of HFSS food advertising varies between 0,8% and 11,2% of total ad revenue for individual TV broadcasters, with an average of 4,56% (based on interviews with TV broadcasters). As a benchmark, for a big Member State, all food and beverages ]* [REF].
v1, p. 51	OPC responses: broadcasters, advertisers, food/beverage industry	Stakeholder input	Consultation	HFSS	In their replies to the public consultation, most broadcasters, advertisers, as well as stakeholders from the food and drinks sector consider that the current rules have been working properly and emphasize the efficiency of self- and co-regulation developed in these areas.
v1, p. 51	OPC responses: public health, MS, regulators	Stakeholder input	Consultation	HFSS	Yet, most stakeholders from the public health sector underline that the current level of protection is not sufficient and the rules should be reinforced. This is supported by 7 Member States and 5 regulators.
v1, p. 51	Study on the exposure of minors to alcohol advertising	Commissioned study	Ecorys et al. (for DG Connect)	Alc	Reinforcing the rules on alcohol advertising would have a positive impact on minors. In particular, a well-adapted watershed in peak time would prevent minors from being exposed to most of the alcohol advertisements. The study on minors' exposure to alcohol advertising showed that minors in most of the 9 selected Member States see most alcohol advertising in TV broadcasts during a certain peak hour. In the Netherlands, where a watershed is in place between 06:00 and 21:00, the average number of impacts for alcohol advertising seen by minors aged 4–14 during peak hour *[between 17:00 and 20:59]* was lower than those in Germany, the UK or the Czech Republic *[6,6 GRP% in NL compared to 36,7 in CZ, 24,1 in DE and 14,1 in the UK. GRP: Gross Rating Points are a measure of advertising impact and GRP% can be interpreted as the total number of times an advertising spot was seen as a percentage of the target group]* which do not apply watersheds.
v1, p. 51	OPC responses: Alcohol Health Alliance, Cancer Research UK, Royal College of Physicians, SHAAP, Alcohol Focus Scotland, European Alcohol Policy Alliance, EPHA, UK Institute of Alcohol Studies.	Stakeholder input	Consultation	Alc	This approach is supported by consumer organisations from the health sector in their replies to the public consultation [REF]. However, one pitfall of such watersheds may be a shift of alcohol advertising just after peak time, at a time when minors, although less numerous, are still watching television quite massively *[for example, the daypart 21:00-23:59 is when minors aged 4–14 in the NL see most alcohol advertisements (27,2 GRP%) as their peak viewing time is between 20:00-21:00 (within the watershed), but there is only a slight decrease in viewing after 21:00. Many NL minors aged 4–14 are still watching TV after 21:00 when alcohol advertisements can be broadcast. In comparison, in FI where a watershed is also applied between 7:00 and 21:00, the daypart 21:00-23:59 is also the period when minors aged 4–14 see most alcohol advertisements, although in lesser proportions (7,5 GRP%). This may be due to the fact that the peak time for minors 4–14 is between 19:00-20:00 and after 21:00 (after the watershed), their viewing has already decreased substantially]*.
v1, p. 51	OPC responses: WFA & public health	Stakeholder input	Consultation	HFSS	The absence of emphasis on the nutritional aspects as being a positive quality of HFSS foods would decrease the appeal of this product for both parents and children. Supporting the development of codes of conduct at EU level in the area of HFSS food advertising to children would have a positive impact on them *[in its reply to the public consultation, the World Federation of Advertisers (WFA) underlines that "the average child under 12 in the EU today sees 88% less ads than in 2005 for products that do not meet EU Pledge nutrition criteria in or around children's programmes". However, most consumer organisations from the health sector criticise the current provisions, highlighting their voluntary character and the fact that the level of protection is not sufficient. In particular, there is no common definition of the nutritional criteria and it should be the role of public authorities to define them]*.
v1, p. 51	OPC responses: public health	Stakeholder input	Consultation	HFSS	However, in the public consultation, several stakeholders from the public health sector criticised the effectiveness of such codes of conduct in protecting minors from exposure to HFSS foods advertising.
v2, p. 10	[REF1] Etats-Unis: et maintenant, moins de coupures de publicité (Feb 2016)	News media	Télérama	AVCC-quant	Nowadays, the TV broadcasting specific rules are too rigid in a world where viewers are likely to switch to alternative offers, in particular without advertising. For example, in the USA where there are no minutage limitations, viewers overwhelmed with TV advertising, turned to other video offers (e.g. video on-demand) thereby disciplining the behaviour of TV broadcasters, who were forced to decrease the amount of advertising on their channels [REF1+2 + 3].
	[REF2] In Dramatic Overhaul, TNT to Cut Ad Load by 50% (Feb 2016)	News media	Adweek	AVCC-quant	
	[REF3] Viacom To Cut Back On Primetime TV Ads Starting In October (Exclusive) (Sept 2015)	News media	Variety	AVCC-quant	
v2, p. 10	Stakeholder input (source unclear)	Stakeholder input	Consultation	AVCC-quant	In the EU, most broadcasters consider that the lack of flexibility of the 12 min and insertion rules and the restrictive character of its exceptions prevent them from maximising their revenues around peak periods.
v2, p. 10	Advertising monitoring (unclear)	Other	Unclear	AVCC-quant	The monitoring of advertising in Member States has indeed shown that this [12 min] rule is regularly breached in a number of Member States.
v2, p. 10	Stakeholder/MS input (source unclear)	Member state; Stakeholder input	Consultation	PPlacement	Some regulators and Member States confirmed that the rules create legal uncertainty for stakeholders, discouraging them to invest in product placement.
v2, p. 43	SQW/Ramboll study	Commissioned study	SQW & Ramboll (for DG Connect)	AVCC-gen	According to regulators, the existing requirements have resulted in administrative costs up to EUR 1.2 million per year per regulatory authority [REF] *[depending on the size of the audiovisual market in the country, the staff costs, the use of external services for monitoring]*.
v2, p. 43	European Advertising Standards Alliance	Stakeholder input	Consultation	AVCC-gen	In the majority of Member States, self- and co-regulation systems are in place in the field of advertising in general. These systems are either funded by membership fees or by a levy system from the industry and their cost ranges from EUR 250 000 to EUR 1 million [REF].
v2, p. 43	OPC responses: advertisers, broadcasters, MS, regulators	Stakeholder input	Consultation	AVCC-gen	In their replies to the 2015 public consultation, advertisers, some broadcasters and several Member States and regulators claim that there is no level playing field between TV broadcasting and other media services, and in particular between TV broadcasters and on-demand service providers. However, a few broadcasters, mainly from the UK, prefer the status quo option.
v2, p. 43	OPC response: BEUC, EURALVA	Stakeholder input	Consultation	AVCC-gen/AVCC-quant	Consumer organisations (including those from the health sector) recognise the relevance of the rules but think that they are neither fair nor effective. Consumer organisations underline that the level of consumer protection should not be lowered [REF] *[in addition EURALVA underlines that the quantitative rules are not satisfactory if not respected by stakeholders]*.
v2, p. 43	Ofcom, Report on UK audience attitudes to the broadcast media, 2014	External report	Ofcom	AVCC-gen	Even if new offers in the market have progressively given consumers the opportunity to switch to services without advertising (see Section 2.2.3), they still have some concerns about excessive advertising on TV [REF] *[in 2014, for example, 57% of UK viewers agreed with the statement “there are already more minutes of advertising in an hour than I am really happy with”. However, viewers also appear to understand the relationship which exists between advertising and the funding of content: 72% of UK viewers questioned in 2014 identified without prompting that advertising represented the primary source of funding for the UK's three main free-to-air commercial television services (ITV/STV/UTV, Channel 4 and Channel 5) which between them account for 24% of UK adult television viewing and just under £1.5bn (€2.1bn) in programme spend].*
v2, p. 43	OPC responses: consumer organisations	Stakeholder input	Consultation	AVCC-gen	They [consumer organisations] also consider that self- and coregulation systems take too long to review complaints while advertising campaigns are fast-paced.
v2, p. 44	SQW/Ramboll study	Commissioned study	SQW & Ramboll (for DG Connect)	AVCC-gen	The incremental costs for TV broadcasting and on-demand service providers of the new provisions [Option A]would be zero [REF] *[advertising scheduling is a core component of broadcast programming and the quantitative rules imposed by the AVMSD are only a small part of a large number of parameters taken into account in TV scheduling strategies aiming at optimising audience and revenue. The costs associated with broadcast programming, including IT costs, are “business as usual”, i.e. costs endured even in the absence of the AVMSD]*.
v2, p. 44	SQW/Ramboll study	Commissioned study	SQW & Ramboll (for DG Connect)	AVCC-quant	There would not be any incremental administrative cost for regulators. Currently, regulators' monitoring and enforcement activities with respect to the 20% limitation amount to up to EUR 1 million [REF] *[based on the current average value for the monitoring of 1 linear provider established in the EU (PPP adjusted) which is derived from a sample of the regulatory costs in 7 MS which can be considered as a representative sample of different approaches to fulfilling regulatory responsibilities with regard to the monitoring and enforcement of the quantitative rules. It is further assumed that regulators focus their regulatory activities on linear services which have more than 0,5 % of the audience share].*
v2, p. 44	SQW/Ramboll study	Commissioned study	SQW & Ramboll (for DG Connect)	Sponsorship	As regards product placement and sponsorship rules, these costs amount respectively up to EUR 2.2 million and EUR 2.1 million per year at EU level [REF].
v2, p. 45	NL, UK, PL, FI and ES	Member state	Consultation	Sponsorship/PPlacement	11 of the Member States that replied to this question support more flexibility but to various degrees. Some refer in particular to sponsorship and product placement rules [REF].
v2, p. 45	FI and its regulator, DE and its regulators and EE	Member state	Consultation	AVCC-quant	Other call also for a deletion of the 20% limitation [REF].
v2, p. 45	Consultation: broadcasters	Stakeholder input	Consultation	Sponsorship/PPlacement	Most broadcasters agree that product placement and sponsorship rules should be clarified and simplified.
v2, p. 45	[REF1] EGTA's report on the costs and benefits of compliance with the Audiovisual Media Services Directive	External report	EGTA	PPlacement	A simplified set of rules on product placement could result in an increase of approximately 10%–15% of product placement revenues [REF1], or in a 4% increase of total advertising revenues in the EU (i.e. potentially additional revenue of EUR 1.2 million) [REF2] *[based on the assumption that current revenues on product placement in Europe capture around 0.1% of total ad revenues and only in some cases might go above 1%, while in countries such as US where the regulations on product placement are very loose or virtually non-existing at all, the market share captured by product placement is around 5%. Such significant direct increase can however hardly be expected in reality (at least short to medium time perspective), mainly due to inherent differences between the EU and the US markets]*.
	[REF2] SQW/Ramboll study	Commissioned study	SQW & Ramboll (for DG Connect)	PPlacement	
v2, p. 45	EGTA's report on the costs and benefits of compliance with the AVMSD	External report	EGTA	Sponsorship	Allowing more flexibility in sponsorship rules would allow broadcasters to generate from 15% to 50% of additional sponsorship revenues [REF].
v2, p. 45	SQW/Ramboll study	Commissioned study	SQW & Ramboll (for DG Connect)	Sponsorship	This could result in more than EUR 441 million increase of total TV advertising spend in the EU (i.e. around 1.5% of current total TV advertising market value) [REF] *[based on an average assumption that of a given range (30%) of an expected increase in revenues from sponsorship activities, as compared to the current estimation that sponsorship captures around 5% (net value) of total TV revenues in their national markets]*.
v2, p. 45	EGTA's report on the costs and benefits of compliance with the AVMSD	External report	EGTA	AVCC-quant	Most broadcasters consider that the insertion rules are no longer relevant or effective. Some argue that because of these rules, schedules are not built around viewers' comfort or advertisers' demand, which is counter-productive. According to the industry, by making the interruption rules more flexible, revenues could increase between 1 and 10 % [REF].
v2, p. 45	Study on defining a new framework for the monitoring of advertising rules under the AVMSD	Commissioned study	SQW & Ramboll (for DG Connect)	AVCC-quant	The only other estimate available is based on the scenario of abolishing interruption rules. In this case, the overall potential revenue gains could amount to 1.35% of advertising revenues coming from cinematographic works and news programmes. This is however a conservative estimate since the parameters for calculation do not take into account different target groups, time slots etc. [REF].
v2, p. 45	EGTA's report on the costs and benefits of compliance with the Audiovisual Media Services Directive	External report	EGTA	AVCC-quant	A shift to a daily limit could generate between a 2% and 15% increase of revenues [REF].
v2, p. 45-46	TF1/GRILLE DES ÉCRANS	Web	TF1	AVCC-gen	For example, over the last six months of 2015 [REF], the gross price of a 30-s advertising spot during prime time on TF1 was EUR 67 330.
v2, p. 46	Nielsen's Advertising and audiences: State of the media Report, 12 May 2014 (p. 14)	External report	Nielsen	AVCC-quant	It is estimated that during important events or programmes, European broadcasters could increase their advertising pressure by a few minutes, from 12 min to 14–15 min *[based on the average minutes of commercials aired* per *hour in the USA* [REF]*]*, taking into account European viewers' lesser propensity to stand long advertising breaks.
v2, p. 46	COMPTE DE RESULTAT SOCIAL (Normes Françaises)	External data	TF1	AVCC-quant	This would mean an increase of around 2,5 min of advertising, i.e. 5 more advertising spots of 30 s, which, all things being equal, could translate for a channel such as TF1 in an increased revenue of 336 650 EUR i.e. theoretically EUR 122 million annually i.e. 10 % of the turnover of the channel in 2014 [REF].
v2, p. 46	MS input: UK & FR	Stakeholder input	Consultation	AVCC-quant	Most commercial broadcasters advocate more flexible [quantitative] advertising rules. However, a few broadcasters (mainly from the UK) deem that rules should remain in their current form in order to keep the advertising market stable. They are supported by some Member States [REF].
v2, p. 46	OPC responses: EPC, EMMA, ENPA, BDZV VDZ, MLE, NMA, VOEZ & ANSO [examples]	Stakeholder input	Consultation	AVCC-quant	The printed press industry claims that more opportunities to advertise on TV could imply changes in advertisers' media mix, which may be to their detriment [REF].
v2, p. 46	OPC responses: SACD, VS, SAA, VdFS [examples]	Stakeholder input	Consultation	AVCC-quant	Due to a relaxation of the interruption rules, there could be more frequent and longer advertising breaks. This may thus affect the integrity of cinematographic works. Several right holders' associations have underlined this in their contributions to the public consultation [REF].
v2, p. 46	[REF1] Etats-Unis: et maintenant, moins de coupures de publicité (Feb 2016)	News media	Télérama	AVCC-quant	However, market developments have led to an increased amount of offerings to which viewers can easily switch, in particular to advertising-free subscription video-on-demand services. This tendency has been clearly observed in the US market [REF1+2 + 3] where, despite the fact that there are no limitations as to the amount of advertising, broadcasters recently use self-restraint in fear of losing audiences.
	[REF2] In Dramatic Overhaul, TNT to Cut Ad Load by 50% (Feb 2016)	News media	Adweek	AVCC-quant	
	[REF3] Viacom To Cut Back On Primetime TV Ads Starting In October (Exclusive) (Sept 2015)	News media	Variety	AVCC-quant	
final, p. 7	Study on the Effectiveness of self and co-regulation in the context of implementing the AVMS Directive (SMART 2014/0054)	Commissioned study	Panteia & VVA (for DG Connect)	AVCC-gen	In general, self-regulatory advertising codes apply to advertising on all media (including TV, on-demand, print media, radio, and online) [REF].
final, p. 12	Survey response: Ofcom	Stakeholder input	Consultation	PPlacement	Some regulators and Member States confirmed that the rules create legal uncertainty for stakeholders, discouraging them to invest in product placement. As a benchmark, in the US, where there is no material regulation of product placement, this format represents almost 5% of the TV ad spend market. In the UK, it represents a share of only 0.1% *[In the US, TV ad spend for 2014 was $69.4 billion with a mid-level forecast of $3.5 billion for product placement. In the UK, the ad market for 2014 was worth £3.56 billion, with product placement capturing £3.5 million of this market*] [REF].
final, p. 12	OPC responses: Broadcasters, alcohol industry, Alcohol Health Alliance, Cancer Research UK, Royal College of Physicians, SHAAP, Alcohol Focus Scotland, European Alcohol Policy, Alliance, EPHA, UK Institute of Alcohol Studies etc [examples]	Stakeholder input	Consultation	Alc	In their replies to the public consultation, most broadcasters, advertisers and stakeholders from the alcohol sector consider that the AVMSD rules on alcohol advertising work well. They point to the efficiency of most self- and co-regulation developed in this area. However, the public health sector underlines that the rules should be reinforced [REF].
final, p. 12	Study on the exposure of minors to alcohol advertising	Commissioned study	Ecorys et al. (for DG Connect)	Alc	The study to measure minors' exposure to alcohol advertising [REF] shows that "on average, a minor in the EU saw 200 alcohol impacts and an adult over 450 during one year (2013)". This means that 1.8% of all advertising seen by minors (under age 18) in 2013 was for alcoholic beverages (as compared to 2.2%. for ads seen by adults). In other words, children are exposed to one impact every two days, and at nearly half the rate of adults.
final, p. 12	Study on the exposure of minors to alcohol advertising	Commissioned study	Ecorys et al. (for DG Connect)	Alc/AVCC-quant	However, one major pitfall of such watersheds may be a shift of alcohol advertising just after peak time, at a time when minors, although less numerous, are still watching television quite massively. As the study on minors' exposure to alcohol advertising showed, when the time is not well adapted, minors may be exposed quite heavily to alcohol advertising just after the watershed [*For example, the daypart 21:00-23:59 is when minors aged 4–14 in the NL see most alcohol advertisements (27,2 GRP%) as their peak viewing time is between 20:00-21:00 (within the watershed), but there is only a slight decrease in viewing after 21:00. Many NL minors aged 4–14 are still watching TV after 21:00 when alcohol advertisements can be broadcast. In comparison, in FI where a watershed is also applied between 7:00 and 21:00, the daypart 21:00-23:59 is also the period when minors aged 4–14 see most alcohol advertisements, although in lesser proportions (7,5 GRP%). This may be due to the fact that the peak time for minors 4–14 is between 19:00-20:00 and after 21:00 (after the watershed), their viewing has already decreased substantially*].
final, p. 12	Study on the exposure of minors to alcohol advertising	Commissioned study	Ecorys et al. (for DG Connect)	Alc/AVCC-quant	Moreover, given the divergences among Member States in peak viewing times for minors [*for example, the peak viewing time for minors aged 4–14 is between 19:00-20:00 in AT, FI and UK but between 22:00-23:00 in ES*], when coupled with the COO principle, watersheds appear less efficient. The applicable watershed would be the one at the country of origin, while minors might be still watching TV in the country of destination.
final, p. 12	Study on the Effectiveness of self and co-regulation in the context of implementing the AVMS Directive (SMART 2014/0054)	Commissioned study	Panteia & VVA (for DG Connect)	Alc	At the same time, the majority of countries have self- or co-regulatory schemes in place [for alcohol]. Some of them are very efficient, while for others, there is scope for improvement [*in particular, the existence of a legislative backstop has been identified as an important success factor in promoting compliance with a self- or co-regulatory code. Graduated sanctions which maintain an element of proportionality are usually considered to be an effective approach in enforcing a scheme* [REF]].
final, p. 12	1st AVMSD Application Report (2012)	EU policy document	European Commission	HFSS	As regards commercial communications for HFSS foods *[Foods High in Fat, Salt and Sugar, defined in the AVMSD as "foods and beverages containing nutrients and substances with a nutritional or physiological effect, in particular those such as fat, trans-fatty acids, salt/sodium and sugars, excessive intakes of which in the overall diet are not recommended"]*, an evaluation of the Platform for Action on Diet, Physical Activity and Health concluded that stakeholders' initiatives in the field of marketing and advertising have made good progress. However, their reach could be further strengthened [REF].
final, p. 13	High Level Group on Nutrition and Physical activity	Web	European Commission (DG SANTE)	HFSS	Marketing of foods and beverages is often a topic of intense discussion during the Platform meetings, and also those of the High Level Group on Nutrition and Physical Activity [REF], with many stakeholders advocating for stricter frameworks (for instance, the High Level Group requested on 28 October 2015 that the WHO Nutrient Profile be used as a basis for dialogue with industry on the issue of food marketing to children).
final, p. 47	European Advertising Standards Alliance	Stakeholder input	Consultation (presumed)	AVCC-gen	In the majority of Member States, self- and co-regulation systems are in place in the field of advertising in general. These systems are either funded by membership fees or by a levy system from the industry and their cost ranges from EUR 250 000 to EUR 1 million [REF].
final, p. 48	OPC responses: advertisers, broadcasters, MS, regulators	Stakeholder input	Consultation	AVCC-gen	In their replies to the 2015 public consultation, advertisers, some broadcasters and several Member States and regulators claim that there is no level playing field between TV broadcasting and other media services, and in particular between TV broadcasters and on-demand service providers. However, a few broadcasters, mainly from the UK, prefer the status quo option.
final, p. 48	OPC response: BEUC, EURALVA	Stakeholder input	Consultation	AVCC-gen	Consumer organisations (including those from the health sector) recognise the relevance of the rules but think that they are neither fair nor effective. Consumer organisations underline that the level of consumer protection should not be lowered *[In addition EURALVA underlines that the quantitative rules are not satisfactory if not respected by stakeholders]* [REF].
final, p. 48	OPC responses: consumer organisations	Stakeholder input	Consultation	AVCC-quant	Even if new offers in the market have progressively given consumers the opportunity to switch to services without advertising (see Section 2.2.3), they [consumer organisations] still have some concerns about excessive advertising on TV [REF] *[in 2014, for example, 57% of UK viewers agreed with the statement “there are already more minutes of advertising in an hour than I am really happy with”. However, viewers also appear to understand the relationship which exists between advertising and the funding of content: 72% of UK viewers questioned in 2014 identified without prompting that advertising represented the primary source of funding for the UK's three main free-to-air commercial television services (ITV/STV/UTV, Channel 4 and Channel 5) which between them account for 24% of UK adult television viewing and just under £1.5bn (€2.1bn) in programme spend].*
final	Ofcom, Report on UK audience attitudes to the broadcast media, 2014	External report	Ofcom	AVCC-quant	
final, p. 48	OPC responses: consumer organisations	Stakeholder input	Consultation	AVCC-gen	They [consumer organisations] also consider that self- and coregulation systems take too long to review complaints while advertising campaigns are fast-paced.
final, p. 49	Study on defining a new framework for the monitoring of advertising rules under the AVMSD	Commissioned study	SQW & Ramboll (for DG Connect)	AVCC-gen	The incremental costs for TV broadcasting and on-demand service providers of the new provisions would be zero [REF] *[advertising scheduling is a core component of broadcast programming and the quantitative rules imposed by the AVMSD are only a small part of a large number of parameters taken into account in TV scheduling strategies aiming at optimising audience and revenue. The costs associated with broadcast programming, including IT costs, are “business as usual”, i.e. costs endured even in the absence of the AVMSD]*.
final, p. 49	Study on defining a new framework for the monitoring of advertising rules under the AVMSD	Commissioned study	SQW & Ramboll (for DG Connect)	AVCC-quant	Currently, regulators' monitoring and enforcement activities with respect to the 20% limitation amount to up to EUR 1 million [REF] *[based on the current average value for the monitoring of 1 linear provider established in the EU (PPP adjusted) which is derived from a sample of the regulatory costs in 7 MS which can be considered as a representative sample of different approaches to fulfilling regulatory responsibilities with regard to the monitoring and enforcement of the quantitative rules. It is further assumed that regulators focus their regulatory activities on linear services which have more than 0,5 % of the audience share].*
final, p. 49	Study on defining a new framework for the monitoring of advertising rules under the AVMSD	Commissioned study	SQW & Ramboll (for DG Connect)	Sponsorship/PPlacement	As regards product placement and sponsorship rules, these costs amount respectively up to EUR 2.2 million and EUR 2.1 million per year at EU level [REF] *[for information, self-regulation organisations' secretariats budget currently range from small (with just one to five members of staff and a budget up to EUR 250 000) to large (up to over 100 members of staff with budgets up to and over EUR 1 000 000) and cover the whole advertising field. SROs' secretariats mainly receive the complaints, gather any necessary information about the complainant and evidence of the advertiser in order to prepare the case for jury. These SROs are either funded by membership fees (18 of them) or a levy system (5) from the industry].*
final, p. 49	Consultation responses: NL, UK, PL, FI and ES	Stakeholder input	Consultation	Sponsorship/PPlacement/AVCC-gen	11 of the Member States that replied to this question support more flexibility but to various degrees. Some refer in particular to sponsorship and product placement rules [REF].
final, p. 49	Consultation responses: FI and its regulator, DE and its regulators and EE.	Stakeholder input	Consultation	AVCC-quant	Other call also for a deletion of the 20% limitation [REF].
final, p. 49	Consultation responses: broadcasters	Stakeholder input	Consultation	PPlacement/Sponsorship	Most broadcasters agree that product placement and sponsorship rules should be clarified and simplified.
final, p. 50	[REF1] EGTA's report on the costs and benefits of compliance with the Audiovisual Media Services Directive	External report	EGTA	Sponsorship/PPlacement	A simplified set of rules on product placement could result in an increase of approximately 10%–15% of product placement revenues [REF1], or in a 4% increase of total advertising revenues in the EU (i.e. potentially additional revenue of EUR 1.2 million) [REF2] *[based on the assumption that current revenues on product placement in Europe capture around 0.1% of total ad revenues and only in some cases might go above 1%, while in countries such as US where the regulations on product placement are very loose or virtually non-existing at all, the market share captured by product placement is around 5%. Such significant direct increase can however hardly be expected in reality (at least short to medium time perspective), mainly due to inherent differences between the EU and the US markets]*.
final	[REF2] Study on defining a new framework for the monitoring of advertising rules under the Audiovisual Media Services Directive	Commissioned study	SQW & Ramboll (for DG Connect)	Sponsorship/PPlacement	
final, p. 50	EGTA's report on the costs and benefits of compliance with the AVMSD	External report	EGTA	Sponsorship	Allowing more flexibility in sponsorship rules would allow broadcasters to generate from 15% to 50% of additional sponsorship revenues [REF].
final, p. 50	Study on defining a new framework for the monitoring of advertising rules under the AVMSD	Commissioned study	SQW & Ramboll (for DG Connect)	Sponsorship	This could result in more than EUR 441 million increase of total TV advertising spend in the EU (i.e. around 1.5% of current total TV advertising market value) [REF] *[based on an average assumption that of a given range (30%) of an expected increase in revenues from sponsorship activities, as compared to the current estimation that sponsorship captures around 5% (net value) of total TV revenues in their national markets]*.
final, p. 50	EGTA's report on the costs and benefits of compliance with the AVMSD	External report	EGTA	AVCC-quant	Most broadcasters consider that the insertion rules are no longer relevant or effective. Some argue that because of these rules, schedules are not built around viewers' comfort or advertisers' demand, which is counter-productive. According to the industry, by making the interruption rules more flexible, revenues could increase between 1 and 10 % [REF].
final, p. 50	Study on defining a new framework for the monitoring of advertising rules under the AVMSD	Commissioned study	SQW & Ramboll (for DG Connect)	AVCC-quant	The only other estimate available is based on the scenario of abolishing interruption rules. In this case, the overall potential revenue gains could amount to 1.35% of advertising revenues coming from cinematographic works and news programmes. This is however a conservative estimate since the parameters for calculation do not take into account different target groups, time slots etc. [REF].
final, p. 50	EGTA's report on the costs and benefits of compliance with the Audiovisual Media Services Directive	External report	EGTA	AVCC-quant	A shift to a daily limit could generate between a 2% and 15% increase of revenues [REF].
final, p. 50, 51	TF1/GRILLE DES ÉCRANS	Web	TF1	AVCC-gen	For example, over the last six months of 2015 [REF], the gross price of a 30-s advertising spot during prime time on TF1 was EUR 67 330.
final, p. 51	Nielsen's Advertising and audiences: State of the media Report, 12 May 2014	External report	Nielsen	AVCC-quant	It is estimated that during important events or programmes, European broadcasters could increase their advertising pressure by a few minutes, from 12 min to 14–15 min *[based on the average minutes of commercials aired* per *hour in the USA* [REF]*]*, taking into account European viewers' lesser propensity to stand long advertising breaks.
final, p. 51	COMPTE DE RESULTAT SOCIAL (Normes Françaises)	External data	TF1	AVCC-quant	This would mean an increase of around 2,5 min of advertising, i.e. 5 more advertising spots of 30 s, which, all things being equal, could translate for a channel such as TF1 in an increased revenue of 336 650 EUR i.e. theoretically EUR 122 million annually i.e. 10 % of the turnover of the channel in 2014 [REF].
final, p. 51	[REF1] Study on defining a new framework for the monitoring of advertising rules under the AVMSD	Commissioned study	SQW & Ramboll (for DG Connect)	AVCC-quant	However, since an increase of the overall advertising volume might trigger a decrease of the advertising price [REF1+2], it is expected that broadcasters will not unduly increase the advertising pressure.
	[REF2] Ofcom, An econometric analysis of the TV advertising market, 2011	External report	Ofcom	AVCC-quant	
final, p. 51	MS input: UK & FR	Stakeholder input	Consultation	AVCC-quant	Most commercial broadcasters advocate more flexible [quantitative] advertising rules. However, a few broadcasters (mainly from the UK) deem that rules should remain in their current form in order to keep the advertising market stable. They are supported by some Member States [REF].
final, p. 51	OPC responses: EPC, EMMA, ENPA, BDZV VDZ, MLE, NMA, VOEZ & ANSO [examples]	Stakeholder input	Consultation	AVCC-quant	The printed press industry claims that more opportunities to advertise on TV could imply changes in advertisers' media mix, which may be to their detriment [REF].
final, p. 51	WORLDPRESSTRENDS by WAN-IFRA	External report	WAN-IFRA	AVCC-quant	However, it should be noted that despite the current limitation, newspaper print advertising in Europe has dropped by 23.1 % between 2010 and 2014 and by 5 % between 2013 and 2014 [REF]. This drop is due to a number of factors, in particular to advertising moving online and to other services. The impact would be limited as it mainly allows broadcasters to better distribute advertising spots during the day.
final, p. 51	[REF1] The Study on defining a new framework for the monitoring of advertising rules under the AVMSD	Commissioned study	SQW & Ramboll (for DG Connect)	HFSS/Alc	Possible codes of conduct at EU level on alcohol and HFSS food advertising would complement activities at national level and are not expected to decrease advertising revenues for EU TV broadcasters *[the Study on defining a new framework for the monitoring of advertising rules under the AVMSD indicates that the share of HFSS food advertising varies between 0,8% and 11,2% of total ad revenue for individual TV broadcasters, with an average of 4,56% (based on interviews with TV broadcasters). The same study indicates that the share of alcohol advertising for individual TV bradcasters varies between 1 and 2,9% of total ad revenue, with an average of 1,99% (based on interviews with TV broadcasters). As a benchmark, for a big Member State, all food and beverages advertising (i.e. broader than HFSS foods and drinks) represented 18% of all TV advertising expenditures. SOURCE: Television International Key Facts 2015, IP Network]* significantly.
	[REF2] Television International Key Facts 2015, IP Network [paywalled]	External data	IP Network	HFSS/Alc	
final, p. 52	OPC responses: SACD, VS, SAA, VdFS [examples]	Stakeholder input	Consultation	AVCC-quant	Due to a relaxation of the interruption rules, there could be more frequent and longer advertising breaks. This may thus affect the integrity of cinematographic works. Several right holders' associations have underlined this in their contributions to the public consultation [REF].
final, p. 52	[REF1] Etats-Unis: et maintenant, moins de coupures de publicité (Feb 2016)	News media	Télérama	AVCC-quant	However, market developments have led to an increased amount of offerings to which viewers can easily switch, in particular to advertising-free subscription video-on-demand services. This tendency has been clearly observed in the US market [REF1+2 + 3] where, despite the fact that there are no limitations as to the amount of advertising, broadcasters recently use self-restraint in fear of losing audiences.
	[REF2] In Dramatic Overhaul, TNT to Cut Ad Load by 50% (Feb 2016)	News media	Adweek	AVCC-quant	
	[REF3] Viacom To Cut Back On Primetime TV Ads Starting In October (Exclusive) (Sept 2015)	News media	Variety	AVCC-quant	
final, p. 52	Study on the impact of marketing through social media, online games and mobile applications on children's behaviour	EU policy document	DG JUST	PPlacement	The deletion of a criterion such as "undue prominence" for product placement would expose viewers to more commercial messages in programmes *[on the impact of product placement on children, see* [REF]].

^1^ Alc = alcohol marketing/consumption; AVCC-gen = audiovisual commercial communications (rules) in general; AVCC-quant = quantitative audiovisual commercial communications (rules); HFSS=HFSS food marketing/consumption; PPlacement = product placement (rules); Sponsorship = sponsorship (rules).

*Abbreviations*: IA = impact assessment; OPC = open public consultation.

**Table:** 

	*Cause*	*Part 1*	*Outcome*
Causal mechanism RSB input	RSB*reviews* IA report and *requests* changes which affect advertising rules	DG Connect*amends* impact assessment report in line with RSB requested changes	Stronger AVCC rules absent from revised/final IA report and legislative proposal
Empirical fingerprints (examples)	RSB first opinion (on first draft IA) is negative, requesting several changes such as greater alignment with evidence base, quantification, and greater emphasis on burden reduction/simplification. It does not request changes to the substance of policy options (in line with RSB remit).	DG Connect amends IA report following negative RSB opinion; removal of stronger alcohol rules embedded in framing around burden reduction/simplification (in line with RSB feedback) but change of substance not evidently intended by RSB. Specifically, the RSB observes in its 2nd opinion that “options have been altered substantially without the consultation of the interservice steering group” and notes that “the reasons why references to the exposure of minors to advertising of HFSS foods and alcohol were withdrawn in both the problem description and the definition of the options, should be clearly explained”.	

	*Cause*	*Part 1*	*Part 2*	*Outcome*
Causal mechanism ‘Evidence-based policymaking’	DG Connect continues to seek to render legislative proposal more evidence-based/align it with BR guidelines on evidence-based policy-making	**[a]**DG Connect*receives/obtains* new evidence relevant to AVCCs	DG Connect*removes* stronger AVCC rules on the basis of unfavourable/insufficient evidence	Stronger AVCC rules absent from revised/final IA report and legislative proposal
		**[b]**DG Connect*revisits/reviews* existing body of evidence relevant to AVCCs		
Empirical fingerprints (examples)	Likely, after RSB requested better alignment with evidence base (see mechanism above).	[a] Unlikely – no new evidence cited regarding alcohol AVCCs in later IA reports. [b] Same sources cited in the first vs later IA report drafts to justify introduction vs non-introduction of stronger alcohol rules. Unclear whether this reflects a re-evaluation or strategic use of evidence.	DG Connect explained removal of stronger rules as follows: “Strengthening the rules on alcohol television advertising has been limited to a re-enforcement of self-co regulation, given that there is not sufficient evidence available to warrant the need to go further” (Annex 1, p. 62, final IA).	

	*Cause*	*Part 1*	*Part 2*	*Outcome*
Causal mechanism Lobbying	Interest groups*become aware* of potential strengthening of AVCC rules, and *mobilise* accordingly	Stakeholders who oppose stricter advertising rules*lobby* DG Connect during IA revision	DG Connect*amends* IA in line with requested changes	Stronger AVCC rules absent from revised/final IA report and legislative proposal
Empirical fingerprints (examples)	A majority of advertising, broadcasting, and food/alcohol representatives not in favour of stronger rules; explicit opposition from a smaller sub-set (as expressed in consultation responses). Groups like World Federation of Advertisers identified stricter rules as concrete possibility.	Disclosure data on meetings with stakeholders (accessed via Integrity Watch EU) during the IA phase, though limited, indicates a clear skew towards commercial actors, with broadcasters enjoying particularly extensive access. In the short period between the submissions of the first and the second IA report draft, relevant officials discussed the AVMSD in meetings with several broadcasting industry groups – namely, the Catalan Audiovisual Council, Sky Group, News Corporation, VAUNET, and the European Broadcasting Union. While it remains possible that interest group pressure (including from the broadcasting industry, which generally did not support stronger rules on food or alcohol advertising) contributed to the final outcome, available evidence is limited.	Removal of stronger rules on alcohol advertising, and the underlying justification, aligns with positions of opposing interest groups, but no direct link established.	

## Appendix A Access-to-document requests

The below requests were made under the EU access-to-documents regime (Regulation (EU) 1049/2001). Documents are returned with personal and sensitive data excluded, precluding the need for anonymisation.

## Appendix C Evidence cited across impact assessments in the context of advertising

*Source type* indicates the origin/nature of the evidence; *topic* refers to the relevance of the claim underlying the evidence citation; context is directly copied from the impact assessment (IA) report versions (v1 = February 2016; v2 = March 2016; final = May 2016).

## Appendix D Causal mechanisms for process tracing

Having established the point in the process where stronger advertising rules for alcohol were removed not only as the preferred option but also from the IA report, what remains unclear is the *why*. Process-tracing, a qualitative method which explores the causal mechanism(s) connecting a known cause and outcome (Beach & Pedersen, 2019; Kay & Baker, 2015), is uniquely suited to interrogate the ‘puzzle’ at hand. We adopt an ‘explaining outcome’ version of the method, aiming to explain a specific outcome (i.e., the removal of stronger rules on alcohol advertising) rather than testing or refining a specific theory. Applying Bayesian logic informally, process tracing starts by establishing *prior* confidence in hypotheses that form part of a causal mechanism, and updates these to *posterior* beliefs in light of available evidence.
